# Total Dairy Consumption Is Not Associated With Likelihood of a First Clinical Diagnosis of Central Nervous System Demyelination

**DOI:** 10.3389/fneur.2022.888559

**Published:** 2022-05-13

**Authors:** Dao Ying Rachel Dieu, Eleanor Dunlop, Alison Daly, Robyn M. Lucas, Yasmine Probst, Lucinda J. Black

**Affiliations:** ^1^Curtin School of Population Health, Curtin University, Perth, WA, Australia; ^2^National Centre for Epidemiology and Population Health, Australian National University, Canberra, ACT, Australia; ^3^Centre for Ophthalmology and Visual Science, University of Western Australia, Perth, WA, Australia; ^4^Faculty of Science, Medicine and Health, University of Wollongong, Wollongong, NSW, Australia; ^5^Illawarra Health and Medical Research Institute, Wollongong, NSW, Australia; ^6^Curtin Health Innovation Research Institute (CHIRI), Curtin University, Perth, WA, Australia

**Keywords:** Ausimmune Study, Australia, nutrition, dairy consumption, multiple sclerosis, diet

## Abstract

**Background:**

The evidence associating consumption of dairy products and risk of MS is contradictory and inconclusive.

**Objective:**

To test associations between dairy consumption and the likelihood of a first clinical diagnosis of central nervous system demyelination (FCD), a common precursor to MS.

**Methods:**

We used data from the 2003–2006 Ausimmune Study, a population-based Australian, multicentre, matched case-control study (272 cases, 519 controls). Total dairy consumption (servings/day) was calculated by summing consumption of milk, cheese and yogurt. Covariate-adjusted treatment effects using augmented inverse probability weighting was used to test for associations with FCD. We conducted sensitivity analyses in the subset of participants who had had a classic first demyelinating event (FDE), defined as a single, first episode of symptoms suggestive of CNS demyelination.

**Results:**

There were no statistically significant associations between total dairy consumption (per one serving/day) and FCD (adjusted OR 1.00; 95% CI 0.93, 1.07; *p* = 0.979). However, yogurt consumption (vs. no yogurt consumption) was associated with an 11% decreased likelihood of FDE (adjusted OR 0.89; 95% CI 0.89, 0.79; *p* = 0.046).

**Conclusion:**

While total dairy consumption was not associated with FCD in this Australian case-control study, yogurt consumption was associated with reduced likelihood of FDE.

## Introduction

The onset of multiple sclerosis (MS) appears to be influenced by a number of factors, including modifiable lifestyle factors, such as low sun exposure and/or low vitamin D status (1, 2), adiposity in childhood to young adulthood (3), and smoking (4). Although diet is potentially a modifiable lifestyle factor for MS onset (5–13), there is no conclusive evidence on what dietary patterns, whole foods and/or nutrients might play a role.

Some studies have shown that dairy consumption is associated with increased risk of MS (14–17), while others have indicated a protective effect (18, 19) or no association (20). Analysis of observational data from the Nurses' Health Study and the Nurses' Health Study II showed no association between intake of dairy products and risk of MS (20). Retrospective data from the same studies did suggest that women who consumed three or more servings of whole milk per day during adolescence (compared to less than one serving/day) had an increased risk of adult-onset MS (17). However, women reporting greater whole milk intake in adolescence were more likely to live in the northern US as adolescents, where vitamin D synthesis by sun exposure is markedly reduced during the winter months, potentially confounding the association.

Several case-control studies in India (15) and Iran (19, 21, 22) have investigated associations between dairy consumption and risk of MS, but with substantial limitations. In a case-control study in India (63 cases, 63 controls) (15), more people with MS compared with controls had a history of daily dairy consumption, but there was no attempt to control for potential confounding. In contrast, a hospital-based Iranian case-control study (536 cases, 399 controls) found that consumption of dairy products was lower in people with MS than controls (21). However, no covariates were considered and the types of dairy products included were not reported (21).

Building on our previous findings that a healthier dietary pattern, a Mediterranean diet, and higher fish and unprocessed red meat consumption were associated with lower likelihood of FCD (5–9), we aimed to test associations between dairy consumption and the likelihood of FCD using data from the Ausimmune Study (23), an Australian, multicenter, matched case-control study investigating environmental risk factors for a first clinical diagnosis of CNS demyelination (FCD).

## Methods

Adults aged 18–59 years were recruited into the 2003–2006 Ausimmune Study from four regions of Australia: Brisbane, Newcastle, Geelong and Western Victoria, and Tasmania. Case participants (*n* = 282) were recruited from the general population and referred to the study by a range of clinicians (e.g., neurologists, ophthalmologists, and general physicians) operating at different tiers in the referral and diagnostic process (23). This was designed to optimize the proportion of incident cases referred to the study, and the response rate of notified cases was >90%. Eligible cases were diagnosed with CNS demyelination for the first time during the course of the study: classic first demyelinating event (FDE), defined as a single, first, episode of clinical symptoms suggestive of CNS demyelination (*n* = 216); primary progressive MS at neurological assessment on study entry (*n* = 18); or, a prior event highly suggestive of CNS demyelination that was neither recognized nor ascribed to demyelination (*n* = 48). A study neurologist confirmed these diagnoses and the date of onset following a full history and neurologic examination. MRI scans taken prior to diagnosis were available for the majority of case participants and were used to indicate date of onset and to determine whether lesions were present.

Control participants (*n* = 558) were randomly selected from the Australian Electoral Roll, for which enrolment is mandatory for citizens aged ≥18 years. Between one and four controls were matched to each case participant based on sex, age (± two years) and study region, with a higher number of controls matched to cases in regions where case numbers were expected to be lower (23). Ethics approval was obtained from the Human Research Ethics Committees of the participating institutions. All participants provided written informed consent.

Dietary intake in the 12 months prior to the study interview was assessed using the Dietary Questionnaire for Epidemiological Studies food frequency questionnaire (FFQ) (*n* = 791) (24). Dairy consumption (grams/day) was reported for milk (full-fat, reduced-fat, and skim), cheese (hard, soft, ricotta, cream cheese, low-fat cheese), and yogurt. We converted intakes in grams/day to servings/day. The amount of milk (full-fat, reduced-fat, and skim), cheese (hard, firm, soft, cream cheese, and low-fat cheese), ricotta/cottage cheese, and yogurt considered as one serving were 1 cup (250), 40, 120 g, and $\frac{3}{4}$ cup (200 g), respectively (25, 26). In line with previous research (27), we did not include butter (commonly classified with fats and oils) (28) or ice-cream (considered an ultra-processed food) (29) in our analyses.

Total dairy consumption (servings/day) was estimated by summing the servings of milk, cheese and yogurt; full-fat dairy consumption by summing the servings of full-fat milk and cheese (including hard, firm, soft, and cream cheese); reduced-fat dairy consumption by summing the servings of low-fat, reduced-fat and skim milk, yogurt and low-fat cheese. Total dairy consumption (servings/day) was also energy-adjusted using the residual method (30). We categorized specific dairy products (full-fat milk, reduced-fat milk, hard/firm cheese, soft cheese, and yogurt) by whether participants consumed the particular product or not (consumption vs. no consumption).

Using self-reported questionnaires, participants indicated highest level of education (up to year 10, year 11 or 12, Technical and Further Education or diploma, or university), history of smoking (yes, no) and history of infectious mononucleosis (yes, no, do not know). Body mass index (BMI) was calculated from height and weight measurements taken at the study interview. Blood samples from participants were used to measure 25-hydroxyvitamin D [25(OH)D] concentration, with seasonal adjustments applied as previously described (1).

Participants with implausible total energy intakes (<3,000 and >20,000 kJ/day) (6) (12 controls; 5 cases) were excluded, leaving a sample of 774 eligible participants. Characteristics of participants whose dairy consumption was below the median were compared with the characteristics of participants whose dairy consumption was at or above the median. Categorical variables were presented as percentage and frequency; normally distributed continuous variables as mean and standard deviation (SD); and non-normally distributed variables as median and interquartile range (IQR). Chi square tests, *t*-tests and Wilcoxon rank sum tests were used to determine statistically significant associations, as appropriate.

Generalized linear models were used to test unadjusted associations between FCD and dairy consumption. Treatment effect models, using augmented inverse-probability weighting (AIPW), were used to explore associations between FCD and dairy consumption (servings/day) for total, full-fat and reduced-fat dairy, and for specific dairy products (full fat milk, reduced fat milk, hard/firm cheese, soft cheese, and yogurt; consumption vs. no consumption). For all AIPW models, the medians of each type of dairy consumption were the cut points for propensity matching (31). Sex, age and study region were covariates for estimation of FCD in the first part of the equation; the second part of the equation incorporated a logit model to show the difference between below and at/above the median dairy consumption as a function of education, BMI, total energy intake, history of smoking, history of infectious mononucleosis and serum 25(OH)D concentrations in association with FCD. Tests for the overlap assumption of the AIPW matched groups (cases and controls) were performed (32). Coefficients from AIPW outcomes were converted to odds ratios (OR) for reporting purposes. Using the same procedures described above, we conducted a sensitivity analysis including only case participants with an FDE and their matched controls. All final models were bootstrapped (500 replicates) with bias corrected standard errors. Data were analyzed using Stata 14 (33).

## Results

Compared with controls, case participants were more likely to have a history of infectious mononucleosis regardless of their dairy consumption (Table 1). Compared to controls, case participants whose total dairy consumption (servings/day) was at or above the median were more likely to have a history of smoking and lower serum 25(OH)D concentrations (Table 1).

**Table 1:** Characteristics of participants included in the current study (*n* = 774; 267 cases, 507 controls) by dairy consumption (servings/day) below the median compared with at or above the median.

	**Dairy consumption (servings/day) below the median**		***p***	**Dairy consumption (servings/day) at or above the median**		***p***
	**Case**	**Control**		**Case**	**Control**	
Sex, % *(n)*						
Male	22.4 (30)	21.0 (53)	0.949	24.1 (32)	23.5 (60)	0.907
Female	77.6 (104)	79.0 (199)		75.9 (101)	76.5 (195)	
Age, mean (SD)	39.5 (9.8)	39.7 (9.8)	0.835	37.5 (9.7)	39.9 (9.5)	0.022
Study region, % *(n)*						
Brisbane (latitude 27°S)	30.6 (41)	32.9 (83)	0.259	36.8 (49)	36.5 (93)	0.405
Newcastle (latitude 33°S)	14.2 (19)	19.1 (48)		12.0 (16)	15.7 (40)	
Geelong (latitude 37°S)	23.9 (32)	25.4 (64)		24.1 (32)	27.5 (70)	
Tasmania (latitude 43°S)	31.3 (42)	22.6 (57)		27.1 (36)	20.4 (52)	
Education, % *(n)*			0.074			0.261
Year 10 or below	21.8 (29)	34.5 (87)		27.8 (37)	30.6 (78)	
Year 11 or 12	18.8 (25)	15.1 (38)		21.1 (28)	13.3 (34)	
TAFE/Diploma	32.3 (43)	25.8 (65)		27.8 (37)	29.0 (74)	
University	27.1 (36)	24.6 (62)		23.3 (31)	27.1 (69)	
Body mass index, median (IQR)	25.9 (6.5)	25.4 (7.9)	0.695	25.9 (8.4)	26.1 (7.3)	0.538
History of smoking, % *(n)*			0.757			0.004
Yes	58.2 (78)	56.6 (142)		63.6 (84)	48.0 (122)	
No	41.8 (56)	43.4 (109)		36.4 (48)	52.0 (132)	
History of infectious mononucleosis, % *(n)*			0.005			0.026
Yes	26.9 (36)	15.1 (38)		28.0 (37)	17.7 (45)	
No	64.9 (87)	80.2 (202)		66.7 (88)	79.2 (202)	
Do not know	8.2 (11)	4.8 (12)		5.3 (7)	3.2 (8)	
Serum 25(OH)D concentrations (mmol/L), mean (SD)	76.7 (29.7)	82.0 (30.7)	0.116	76.1 (30.2)	82.9 (30.3)	0.039

*The following variables had missing data: education (1 case); body mass index (1 case, 3 controls); serum 25(OH)D concentrations (8 cases, 30 controls)*.

*SD, standard deviation; 25(OH)D, 25-hydroxyvitamin D; IQR, interquartile range; TAFE, Tertiary and Further Education*.

All AIPW models met the overlap assumption and tests for balance between cases and controls. Table 2 shows unadjusted, covariate-adjusted and energy-adjusted models for total dairy consumption and the likelihood of FCD and FDE. There were no statistically significant associations. We also found no statistically significant associations between full-fat dairy consumption or reduced-fat dairy consumption and FCD or FDE (Table 3). Similarly, there were no statistically significant associations between consumption of specific dairy products and likelihood of FCD (Table 3). There was no statistical difference between cases and controls in yogurt consumption in unadjusted models; however, there was an 11% decreased likelihood (OR 0.89; 95%CI: 0.79, 1.00) of FDE with yogurt consumption compared to no yogurt consumption in covariate-adjusted and energy-adjusted models (Table 3).

**Table 2:** AIPW^a^ models showing associations between the likelihood of FCD and FDE with total dairy consumption.

	**OR^**2**^ (95% CI)**	***p***
**FCD (*****n*** **=** **732; 257 cases, 475 controls)**		
Unadjusted (per one serving/day)	1.00 (0.89, 1.12)	0.946
Adjusted for covariates, including total energy intake (kJ/day) (per one serving/day)	1.01 (0.94, 1.08)	0.791
Adjusted for covariates, excluding total energy intake (kJ/day) (per one serving/day)	1.00 (0.93, 1.07)	0.979
Adjusted for covariates, excluding total energy intake (kJ/day) (per one energy-adjusted serving/day)^b^	1.05 (0.97, 1.13)	0.211
**FDE (*****n*** **=** **553; 200 cases, 353 controls)**		
Unadjusted (per one serving/day)	1.05 (0.93, 1.19)	0.411
Adjusted for covariates, including total energy intake (kJ/day) (per one serving/day)	1.05 (0.97, 1.15)	0.234
Adjusted for covariates, excluding total energy intake (kJ/day) (per one serving/day)	1.04 (0.96, 1.13)	0.284
Adjusted for covariates, excluding total energy intake (kJ/day) (per one energy-adjusted serving/day)^b^	1.06 (0.98, 1.16)	0.142

^a^*Based on treatment effects model using AIPW. The AIPW coefficients were converted to odds ratios using an exponential function. The first equation used sex, age and study region as covariates in a logit model; the second equation used the median of dairy consumption (either servings/day or energy-adjusted servings/day) with education, body mass index, history of smoking, history of infectious mononucleosis and serum 25-hydroxyvitamin D (25(OH)D) concentrations as covariates in a logit model (with and without total energy intake, as indicated)*.

b *With total dairy consumption energy-adjusted using the residual method*.

*AIPW, augmented inverse-probability weighting; FCD, first clinical diagnosis of central nervous system demyelination; FDE, classic first demyelinating event*.

**Table 3:** AIPW^a^ models showing associations between the likelihood of FCD and FDE with full-fat dairy consumption, reduced-fat dairy consumption, full-fat milk, reduced-fat milk, hard/firm cheese, soft cheese and yogurt.

	**Unadjusted OR (95% CI)**	***p***	**Adjusted for covariates, including total energy intake (kJ/day) OR (95% CI)**	***p***	**Adjusted for covariates, excluding total energy intake (kJ/day) OR (95% CI)**	***p***
**FCD (*****n*** **=** **732; 257 cases, 475 controls)**						
By fat content						
Full-fat dairy (per one serving/day)	1.02 (0.92, 1.13)	0.750	1.00 (0.93, 1.07)	0.977	1.01 (0.94, 1.08)	0.843
Reduced-fat dairy (per one serving/day)	0.98 (0.88, 1.09)	0.736	1.03 (0.96, 1.11)	0.481	1.03 (0.96, 1.11)	0.434
By dairy product						
Full-fat milk (consumption vs. no consumption)	1.02 (0.90, 1.15)	0.771	0.99 (0.91, 1.06)	0.698	0.98 (0.91, 1.05)	0.554
Reduced-fat milk (consumption vs. no consumption)	1.13 (0.99, 1.28)	0.082	1.03 (0.97, 1.11)	0.355	1.04 (0.97, 1.12)	0.305
Hard/firm cheese (consumption vs. no consumption)	1.14 (0.81, 1.61)	0.462	1.05 (0.97, 1.14)	0.183	1.04 (0.96, 1.12)	0.313
Soft cheese (consumption vs. no consumption)	0.63 (0.22, 1.80)	0.387	0.95 (0.85, 1.07)	0.447	0.96 (0.86, 1.06)	0.447
Yogurt (consumption vs. no consumption)	0.73 (0.49, 1.10)	0.132	0.92 (0.83, 1.02)	0.119	0.92 (0.84, 1.02)	0.121
**FDE (*****n*** **=** **553; 200 cases, 353 controls)**						
By fat content						
Full-fat dairy (per one serving/day)	1.05 (0.93, 1.17)	0.451	1.00 (0.91, 1.09)	0.947	1.00 (0.92, 1.08)	0.969
Reduced-fat dairy (per one serving/day)	1.00 (0.89, 1.13)	0.969	1.03 (0.95, 1.12)	0.441	1.03 (0.95, 1.12)	0.414
By dairy product						
Full-fat milk (consumption vs. no consumption)	1.04 (0.92, 1.18)	0.519	0.97 (0.89, 1.06)	0.521	0.97 (0.89, 1.05)	0.463
Reduced-fat milk (consumption vs. no consumption)	1.17 (1.01, 1.36)	0.035	1.05 (0.97, 1.14)	0.263	1.05 (0.97, 1.14)	0.248
Hard/firm cheese (consumption vs. no consumption)	1.19 (0.83, 1.71)	0.357	1.03 (0.94, 1.13)	0.572	1.02 (0.93, 1.12)	0.666
Soft cheese (consumption vs. no consumption)	0.81 (0.28, 2.34)	0.694	0.94 (0.83, 1.08)	0.395	0.94 (0.83, 1.08)	0.399
Yogurt (consumption vs. no consumption)	0.64 (0.40, 1.03)	0.064	0.89 (0.79, 1.00)	0.046	0.89 (0.79, 1.00)	0.043

^a^*Based on treatment effects model using AIPW. The AIPW coefficients were converted to odds ratios using an exponential function. The first equation used sex, age and study region as covariates in a logit model; the second equation used the median of dairy consumption by fat content or by dairy product with education, body mass index, history of smoking, history of infectious mononucleosis and serum 25-hydroxyvitamin D (25(OH)D) concentrations as covariates in a logit model (with and without total energy intake, as indicated). AIPW, augmented inverse-probability weighting; FCD, first clinical diagnosis of central nervous system demyelination; FDE, classic first demyelinating event*.

## Discussion

We found no association between any type of dairy consumption and the likelihood of FCD or FDE. However, yogurt consumption (compared with no yogurt consumption) was associated with a small decreased likelihood of FDE. We chose the more statistically powerful treatment effects model based on the AIPW approach (34) rather than conditional logistic regression to examine the associations between FCD and dairy consumption. This approach maximized the chance of finding any significant associations, while the bootstrapping produced robust and bias corrected confidence intervals, decreasing the likelihood of false negative or positive associations.

In our previous study using data from the United States' MS Sunshine Study (602 cases, 653 controls), we found that higher consumption of yogurt between childhood and young adulthood (ages 6–20 years) was protective against adult-onset MS (35). Similarly, an Iranian case-control study (547 cases, 1,057 controls) found that higher consumption of yogurt during adolescence was associated with reduced risk of adult-onset MS (22), while another Iranian case-control study in Iran (660 cases, 421 controls) showed that higher consumption of milk and yogurt was associated with decreased likelihood of MS (19). All three studies reported type of dairy (e.g., milk, yogurt, cheese) and frequency of consumption, but portion size was considered in only one study (22). Nevertheless, those findings are consistent with our current findings in relation to yogurt and FDE.

The association between yogurt and FDE may relate to the probiotic properties of yogurt. Yogurt is often a carrier of probiotics—live microorganisms (e.g., *Lactobacillus acidophilus, Bifidobacteria* spp.) which, if consumed in adequate amounts, may have health benefits (36). Probiotics potentially modulate the gut microbiota and, given that the gut microbiota may modulate the immune response, gut microbiota may play a role in the pathogenesis of MS (37). As the FFQ used did not collect information on the live culture content or probiotic potential of yogurts consumed, it is uncertain whether the potential links between probiotics, gut microbiota, and inflammation could explain the inverse association between yogurt and FDE seen in our study. Furthermore, it is uncertain why this association was found for FDE and not FCD.

Various mechanisms have been proposed to support either a protective or deleterious effect of dairy consumption on risk of MS, including a protective effect of calcium. There is some evidence from animal studies that low calcium intake may adversely affect myelin lipid synthesis, which has been demonstrated in mouse models (38). However, calcium intake was not associated with MS risk using data from the NHS and NHSII (39). Dairy products, particularly milk, are fortified with vitamin D in some countries (40), and are important contributors to dietary vitamin D intakes in the US and Canada (41, 42). However, the studies on MS risk from the NHS and NHSII showed no statistically significant association between vitamin D intake from food and risk of MS (43). Neither India nor Iran, the settings for the aforementioned case-controls studies (15, 19, 21), appeared to have systematic vitamin D-fortification of dairy products at the time those studies were conducted (44, 45). Although fortification with vitamin D of certain dairy foods is permitted in Australia (46), few products are vitamin D-fortified in practice. Therefore, even if dairy consumption was protective against MS through added vitamin D, this would not likely be observed in our Australian cohort.

A deleterious effect of the milk protein, butyrophilin, has been implicated in MS risk. Two studies have shown molecular mimicry with myelin oligodendrocyte glycoprotein (47, 48), triggering an encephalitogenic T cell response in experimental autoimmune encephalomyelitis (EAE), an animal model for MS (48). However, any link between dairy products, butyrophilin, and myelin oligodendrocyte glycoprotein, remains speculative (47) and the role of encephalitogenic T cells in MS in humans is not well-understood (49).

A strength of our study was the well-characterized sample of people with early MS, with an incident case-control design. However, participants were living in Australia and predominately of European descent; hence, our findings may not be generalizable to other populations. Although we cannot rule out potential residual confounding by other demographic or lifestyle characteristics, such as socioeconomic status, that may be associated with consumption of dairy products, various lifestyle characteristics (e.g., BMI, alcohol intake and physical activity) were not associated with risk of FCD in previous analysis of data from the Ausimmune Study (50). The food consumption data used in this study are subject to the acknowledged limitations of self-reported dietary intake, such as recall bias and measurement error.

In conclusion, we found no association between the likelihood of FCD and total dairy consumption in this Australian population. We did find a small decreased likelihood of FDE associated with yogurt consumption. Dairy foods are an important source of calcium, vitamin D (in some countries), and other vitamins and minerals. Given that many diets promoted for people with MS exclude dairy products, investigating the effect of dairy consumption on MS disease progression, as opposed to onset, is warranted to help develop evidence-based dietary guidance for people with MS.

## Data Availability Statement

The data analyzed in this study was obtained from the Ausimmune Study, the following licenses/restrictions apply: The data can be made available for analysis with a collaborative agreement with the Ausimmune Investigator Group. Requests to access these datasets should be directed to Professor Anne-Louise Ponsonby, annelouise.ponsonby@florey.edu.au.

## Ethics Statement

The studies involving human participants were reviewed and approved by Barwon Health Research and Ethics Advisory Committee (ref 03/46), Ballarat Health Services and St John of God Health Services Committee, Royal Brisbane and Women's Hospital and Health Service District Office of the Human Research Ethics Committee (ref 2003/093), The University of Queensland Medical Research Ethics Committee (ref 2003000253), The Princess Alexandra Hospital Human Research Ethics Committee (ref 2004/059), The Queensland Institute of Medical Research Human Research Ethics Committee [ref H0511-061 (P950)], Hunter Area Research Ethics Committee (ref 03/06/11/3.07), Southern Tasmania Heath and Medical Research Ethics Committee (ref H7436), The Australian National University Human Research Ethics Committee (ref 2002/111). The patients/participants provided their written informed consent to participate in this study.

## Author Contributions

LB had primary responsibility for the final content and designed the study. DD, ED, and LB wrote the manuscript. AD analyzed the data and interpreted the results. RL, YP, AD, and Ausimmune Investigator Group provided critical revision of the manuscript for important intellectual content. All authors read and approved the final version of the manuscript.

## Funding

Funding for the Ausimmune Study was provided by the National Multiple Sclerosis Society of the United States of America (NMSS RG 3364A1/2) and the National Health and Medical Research Council of Australia (313901). LB is supported by MS Western Australia (MSWA), an MS Australia Postdoctoral Fellowship, and a Curtin University Research Fellowship. RL is supported by a National Health and Medical Research Council of Australia Senior Research Fellowship. AD is supported by MSWA. Funding bodies had no role in the design or conduct of the study, collection, management, analysis or interpretation of data or preparation, and review or approval of the manuscript.

## Conflict of Interest

The authors declare that the research was conducted in the absence of any commercial or financial relationships that could be construed as a potential conflict of interest.

## Publisher's Note

All claims expressed in this article are solely those of the authors and do not necessarily represent those of their affiliated organizations, or those of the publisher, the editors and the reviewers. Any product that may be evaluated in this article, or claim that may be made by its manufacturer, is not guaranteed or endorsed by the publisher.
